# Supplementary material for: Ethno-veterinary practice for the treatment of animal diseases in Neelum Valley, Kashmir Himalaya, Pakistan
Source: PLoS One. 2021 Apr 30;16(4):e0250114. doi: 10.1371/journal.pone.0250114 (PMC8087047; doi:10.1371/journal.pone.0250114)
Supplement: S1 File — (DOCX) [file pone.0250114.s001.docx]

**Annexure – I QUESTIONNAIRE NO: 1**

**Documentation of Ethnobotanical Knowledge about Medicinal Plants**

Locality of the Medicinal Plants/ Collection site:_______________________

Date:________ Name of the collector:____________________

Age of the collector:__________ Education level of the collector:____________

Local name of the plant species collected:_______________________________

Scientific name of the species collected:_________________________________

Habitat of the species collected:________________________________________

Uses of the plant species:_____________________________________________

Quantity harvested per day/ month/year:_________________________________

Who collect the plant (Child.Woman/Man), why?_________________________

Ethnic group (Mughal/Chaudhary/Raja /Awan/Nomads/other___________

Is it sold?: ____________________________________

Quantity sold per day/month/year:___________________________________

To whom is it sold?_________________ Price/Kg: _____________________

Is the plant perceived by the informant to be abundant/common/rare?_________

Changes in abundance of the plant for the last 10 years (more abundant/same/rare)

________________________________________________________________

Are methods used to regenerate or otherwise actively manages the plant?_____________ What? _______________________

What kinds of tools are being used for harvesting?_________________________

What kinds of traditional methods are being used for the processing of medicinal plants after harvesting?: ______________________________________________

Are these traditional methods bringing results? Yes/No:____________________

If, No, Then what are the problems faced in this regard?: _________________

**Annexure – II QUESTIONNAIRE NO: 2**

**Medicine, Fodder, Fuel wood, Timber, Vegetable, Food, Fruits, Herbal tea, Veterinary uses, Joinery works, Poisonous, Spices/Condiments informations**

Local practices of treatment (Herbal/ English medicines)____________________

Use of plants as medicines (names) ____________________________________

Use of plants as fuel wood (names) _____________________________________

Use of plants as timber (names) _______________________________________

Use of plants as vegetable (names) _____________________________________

Use of plants as Veterinary medicines (names) ____________________________

Use of plants as Spices & Condiments (names) ____________________________

Local fruits and their uses (names) _____________________________________

Fruit trees grown or not/ if grown then what species are grown_______________

________________________________________________________________

Wild plant species in the land_________________________________________

Whether used by the local people or not__________________________________

**Livestock**

Having livestock or not______________________________________________

Preference for kind of livestock (Buffalo, Cow, Goat, Sheep, Donkey, Horse)

__________________________________________________________________

Why you prefer this kind of livestock?__________________________________

Number of livestock_________________________________________________

Annual income from livestock_________________________________________

**Fodder**

How do you feed your livestock?________________________________________

Where do you graze your livestock (Forest, Rangeland, Agriculture fields, Others)

_________________________________________________________________

Quantity of fodder species used________________________________________

Fodder consumed by Goat/ Buffalo/ Cow/ Sheep etc.________________________

Fodder species Stored for winter_______________________________________

How long cattles graze open in the pastures_______________________________

**Fuel wood**

Present Sources of wood-fuel (forest, agriculture land etc.)___________________

What species are used? (Conifers or broad leaves) __________________________

In what quantity____________________________________________________

During Summer daily use (kg)_________________________________________

During Winter daily wood use (Kg)_____________________________________

What is preferred species during summer? _______________________________

What is preferred species during winter? _________________________________

Collected from reserved forest or agricultural lands _______________________

What is the method of transportation? ____________________________________

Who collect wood-fuel (Men, Women, Children)___________________________

**Timber**

Your annual timber needs (Construction, joinery works, Others)_______________

Present sources of timber (Forest, Others)________________________________

Past sources of timber. ______________________________________________

Do you sell timber?__________________________________________________

Where you sell timber?_______________________________________________

Do you purchase timber?______________________________________________

What is the rate/Kg or rate / jeep/truck? __________________________________

**Annexure-III: QUESTIONNAIRE NO. 3**

**Questionnaire for Conservation Survey/Status**

Date:_______________________ Name of plant collector:_____________________

Age: __________Gender:__________ Education:______________

Locality:____________________

**Information pertinent to Conservation Status of the Flora**

Local Name of Plant Collected:__________ Collection Time:______________

Part Collected: _____________ Collection Method:___________________________

Uses of plant: _____________ Locally:__________ Market:_________

Present Availability Status: ______________________________________________

Availability Status over the past 10 years: Persistent/ Increased/ Decreased. ___________

Reasons:_________________________________________________________________

Any Conservation efforts by Collector/Community:_____________________________

Name of the most threatened plants Species:___________________________________

Any Extinct Species: Yes/No If Yes, then which one an why:______________________

Other observations:_______________________________________________________
