# Supplementary figures and images for: Ethno-veterinary practice for the treatment of animal diseases in Neelum Valley, Kashmir Himalaya, Pakistan
Source: PLoS One. 2021 Apr 30;16(4):e0250114. doi: 10.1371/journal.pone.0250114 (PMC8087047; doi:10.1371/journal.pone.0250114)

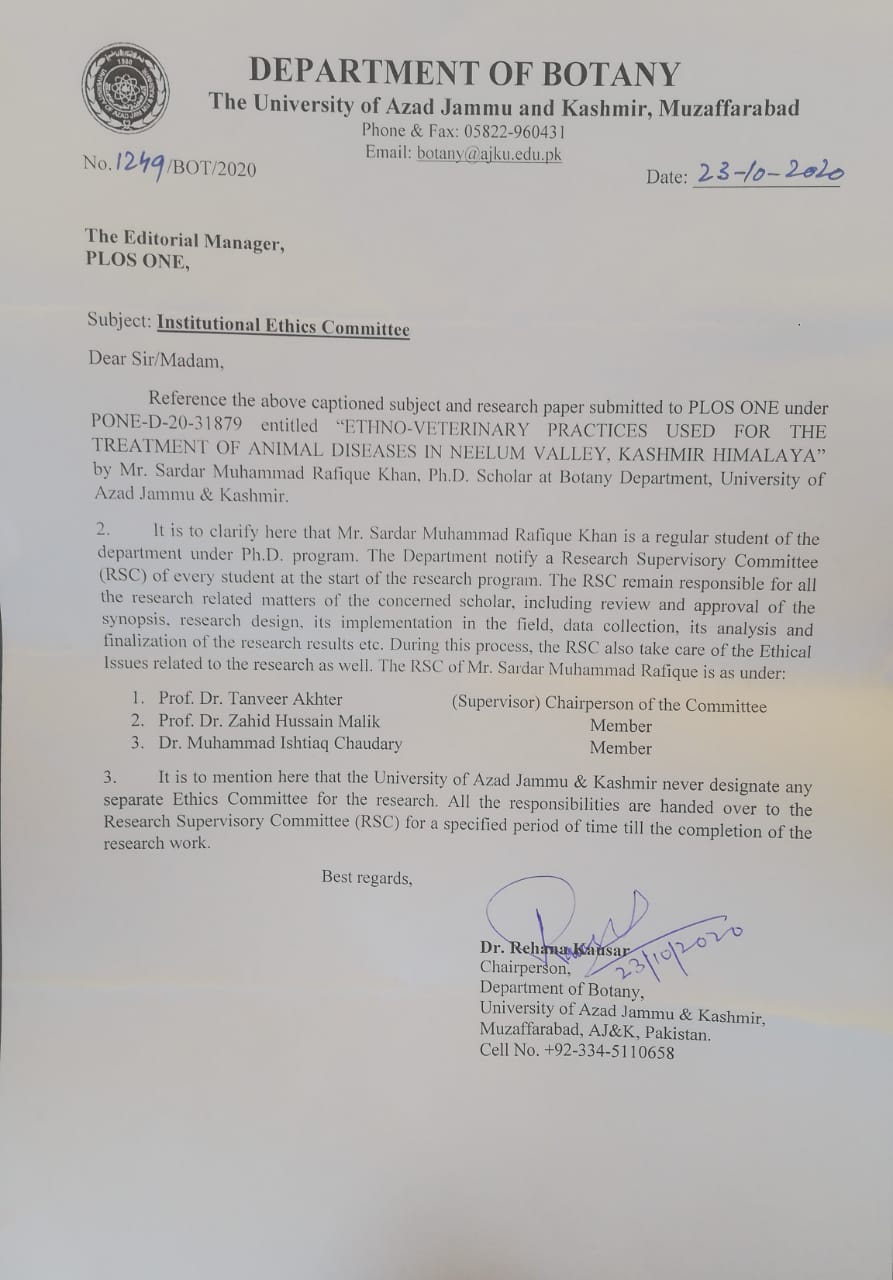

Supplement: S1 Fig — (JPG) [file pone.0250114.s002.jpg]
