# Supplementary material for: Ethno-veterinary practice for the treatment of animal diseases in Neelum Valley, Kashmir Himalaya, Pakistan
Source: PLoS One. 2021 Apr 30;16(4):e0250114. doi: 10.1371/journal.pone.0250114 (PMC8087047; doi:10.1371/journal.pone.0250114)
Supplement: Attachment — Submitted filename: Review comment.docx [file pone.0250114.s003.docx]

**Review comments of manuscript PONE-D-20-31879**

The manuscript presented the ethnoveterinary knowledge of plants, which is used by the local inhabitants in Neelum Valley, Kashmir Himalaya for various disease of animals. The most important issue is that the manuscript was found to have a very significant degree of overlap with existing articles. I have identified a large sections in the manuscript that seem to be taken verbatim from other sources and without giving proper references to these sources and without identifying the text as a word by word citation.

I don’t recommend its publication in the form in which it is presented my recommendation is reject and resubmit, I therefore kindly request the authors to provide a substantially revised version of this manuscript to address these concerns and some others suggestions, which is annotated directly in PDF. Note that not all matches in your report will be of concern, in particular commonly found phrases in manuscripts will be highlighted regardless and can be ignored (e.g., acknowledgments section and technical language in method section).

Some others basics missing which I have found and that in each case are necessary for a sound publication as:

The language is not suitable for publication; the manuscript needs a major English revision before being submitted. Formatting of the paper is very poor. Check the manuscript thoroughly for spelling mistake and grammar.

The abstract is not clear and with many serious grammar mistakes.

Your introduction needs more detail. I suggest that you improve the description in the last lines to provide more justiﬁcation for your study (speciﬁcally, you should expand upon the knowledge gap being ﬁlled). Also, write the clear objectives of the study.

The methodology section is ppoorly organized. No information is available about how the study was design etc.

The results is very poorly presented.

Discussion is not result based, also need improvement grammatically. The whole section has to be revised not only for the language, but also for its structure.

Formatting of tables are in poor order. Please provide voucher specimens number in the table.

The conclusion is overstated, and the major results of the research were not reported.

Critically check your references, some references have missing volume number and page number. Follow Plos One journals guidelines for all references.

There are more annotations in the Pdf, which should be considered by the authors.
