# Supplementary material for: Ethno-veterinary practice for the treatment of animal diseases in Neelum Valley, Kashmir Himalaya, Pakistan
Source: PLoS One. 2021 Apr 30;16(4):e0250114. doi: 10.1371/journal.pone.0250114 (PMC8087047; doi:10.1371/journal.pone.0250114)
Supplement: Attachment — Submitted filename: 2020-Ethno-veterinary Paper _Editor Comments.docx [file pone.0250114.s006.docx]

**ETHNO-VETERINARY PRACTICES USED FOR THE TREATMENT OF ANIMAL DISEASES IN NEELUM VALLEY, KASHMIR HIMALAYA**

**ETHNO-VETERINARY PRACTICES USED IN NEELUM VALLEY, KASHMIR HIMALAYA**

Sardar Muhammad Rafique Khan^1^, Tanveer Akhter^1^ and Mumtaz Hussain ^2^

^1^Department of Botany, University of Azad Jammu & Kashmir, Muzaffarabad, Pakistan

^2^ Hazara University, Mansehra, Pakistan.

**Abstract**

Plant species are not only used as fodder or forage but also contribute substantially to the treatment of various health disorders, particularly in livestock. The current study is the first quantitative ethnobotanical on ethnoveterinary uses of medicinal plants conducted in the Upper Neelum Valley of Azad Jammu & Kashmir. The methods employed during the present study were surveys, conducted during different seasons of the year 2012-15, in various localities inhabited by the local communities. Preferably elderly and experienced members of the tribes, locally known as `Budhair' (aged) above the age of forty, were interviewed. They were often accompanied to the field for the identification of plant species those were used and their preferred habitats. Information pertaining to cure different ailment of animals was collected from 126 informants through semi-structured interviews, group discussion and field walks. This was further analyzed for the authenticity through ethnobotanical indices. Traditional ethno-veterinary medicinal uses of 39 species, belonging to 31 genera and 21 families were documented which were used by the indigenous communities of Kashmir Himalaya for curing 21 different diseases/ailments of 7 different types of livestock. Out of the totals 29 major ailments, 8 are reported to be cured without involvement of plants but treated traditionally. The highest number of ethno-medicinal plants were recorded from family Polygonaceae followed by Crassulaceae, Asteraceae and other families. Roots were the most commonly used part of the plant for the preparation of ethno-veterinary medicines followed by aerial parts. The highest frequency of citation and relative frequency of citation was found for *Saussurea lappa* (41/7.32), followed by *Rumex acetosa* (37/6.61), *Rumex nepalensis* (36/6.43), *Thymus linearis* (28/5.0) and *Angelica cyclocarpa* (28/5.0). The highest use value was recorded for *Saussurea lappa* (0.33), followed by *Rumex acetosa* (0.29), *Rumex nepalensis* (0.29), *Thymus linearis* and *Angelica cyclocarpa* (0.22 each). Inhabitant communities of the rich floristic study area are dependent on medicinal plants for ethnoveterinary practices to cure livestock diseases. Knowledge about the traditional medicinal system is restricted to the herders, farmers and elder community member. The current study has an important contribution towards the preservation of indigenous plants-based knowledge from extinction. The phytochemical and pharmacological investigations are suggested to isolate the active compound and testing the in-vitro or in-vivo efficiency of the abovementioned plants against the targeted veterinary diseases. Awareness among the inhabitants of the area can be further helpful in transfer and preservation of this important knowledge and conservation of important natural flora.

**Key words:** Livestock, Ethno-veterinary uses, Use value, Kashmir Himalaya

**Introduction**

The importance of medicinal plants has turned around the world to natural medicines. The use of these medicine has emphasized on the importance of seeking knowledge about the medicinal plant species those traditionally were used by the indigenous communities of the mountain areas. The growing evidences indicate strong relationship between sustainable use of the biodiversity and ethnic knowledge [1]. Ethnic knowledge, which is time tested and supplemented with the latest scientific insights, can offer new models of economic development, which are socially acceptable and eco-friendly [64]. Documentation of folk knowledge has gained substantial importance around the world especially with the ratification of the Nagoya Protocol in order to maintain cultural heritage [2].

By virtue of its topographic variations spanning from valley floor through terraced lands and dense forests, up to snow-capped alpine peaks, the Kashmir Himalaya, hovering at the western tip of the Himalayan Biodiversity Hotspot, supports rich biodiversity of great scientific curiosity and promising economic benefits [3]. Mosaic of diverse niches -a byproduct of the habitat heterogeneity and the microclimatic variation along the altitudinal gradient display a bewildering floristic diversity [4]. The communities in the mountain are semi-nomadic tribes mostly inhabiting the hilly areas of the Kashmir Himalaya. They mainly depend on the livestock rearing and subsistence agriculture for their livelihood. Living on the edges of the mountains, they traditionally graze their livestock such as cows, bulls, buffaloes, goats, sheep, horses, etc. throughout the landscape in the region [5].

From decades, the inhabitants have learned and practiced the medicinal usage of plants growing in their close vicinity treating various ailments of their livestock in the need of hour. It is estimated that medicinal plants for several centuries have been widely used as a primary source of prevention and control of livestock diseases [6]. This precious ancient wisdom has usually been disseminated through the word of mouth (orally) from one generation to the other, having no black and white record. The transmission of traditional knowledge through oral methods is however unreliable and authentication through documentation is required [7]. Therefore, it is dire need of the time to document it on scientific lines before it disappears due to rapid socioeconomic, environmental, technological changes and as a result of loss of the cultural heritage under the guise of civilization**.** The only solution is documentation and conservation through systematic scientific studies before it is lost forever. In fact, interest/importance of such use in the veterinary sector has resulted primarily from the increasing cost of livestock, besides the maintenance and introduction of new technology in the production of veterinary medicines and vaccines. Ethnoveterinary medicines have the ability to fight different kinds of animal disorders [8] and several advantages are synthetic drugs. Ethnoveterinary medicines are easy to use, cheap and readily available [9].

During the last century, various studies have been carried out to document the ethno-medicinal uses of the plant species growing in the region. However, research efforts made so far, have been restricted to the chronicling of the medicinal plants used for the treatment of human ailments [10-18, 4, 19-20]. Very rarely, the ethno-veterinary medicinal uses of the floral diversity have been investigated [21-28]. Some workers have documented the indigenous ethno-veterinary practices in different parts of Pakistan [29-32, 19, 33-34], still documentation for the conservation of this well tested knowledge is required to be done in many parts of the country. The main objective of the present research, in this backdrop, was to fill the knowledge gaps in this important sub-area of cultural biodiversity directly relevant to the livelihood of the indigenous communities.

There are very limited livelihood opportunities available for the people of Neelum Valley and most of the pastoralists in the mountain part of AJ&K and the farmers in the high fertile lands. Livestock plays a pivotal role as it provides farmyard manure, rural transport, milk, meat and source of entertainment in the sports like Polo and also has major role in rural economy by providing income and employment to small hold farmers and poor people of the society. Easily accessible and available Ethnoveterinary medicinal plants provide a cheaper source for treatment of various diseases. The hardship that people face is the seasonal availability of certain plants, to overcome this issue, farmers have acquired different ways to preserve them for off-season uses. In these communities, the modern veterinary health curative system is inadequate, therefore the inhabitants utilize traditional ethnoveterinary medicinal health system for health care. The economic condition of the farmers also restricts them to the use of modern allopathic drugs, which ultimately leads to poor livestock production and financial losses due to poor health of animals. Under such circumstances, Ethnoveterinary medicines can be promoted as an alternative drug and it can help in alleviation of the poverty by empowering the people to make use of their own resources for the treatment of their livestock.

**Materials and Methods**

**Study area:** Natural geomorphological features of Pakistan range from the icy/ snowcapped peaks of Himalaya and other mountain ranges in the north, the sandy beaches and mangrove swamps in south; allowing different landscapes and climates with variety of flora and fauna. This study was conducted in District Neelum of Azad Jammu & Kashmir (AJ&K), which is a hilly area with rugged topography, located in the extreme north of the AJ&K (Fig. 5). Total area of the district Neelum is 3621 Sq. kms with a population of 1.96 million [35]. Neelum Valley is located at 74°- 24′ - 50″ to 74° - 31′ - 50″ longitude and 34° - 50′ - 40″ to 35° latitudes. It is home to different ethnic groups like; *Mughal, Chaudhry, Butt, Pire, Wani, Syed, Malik, Turks, Khawaja, Rajput* etc. Leading distinctive features of District Neelum are its mountain ranges, natural lakes, waterfalls and the valleys. The valley is rich in the floral diversity. Documentation was carried out in three sub-valleys of the district Neelum i.e., Surgan, Shounther and Guraize Valley and in a most populated town area Kel (Fig. 1).

**Figure 1: Map of the stud y Area**

**Ethnoveterinary field work and interviews**

**Data collection:** Before starting the process of field work, the research methodology / protocol was finalized followed by the seeking formal approval from the Supervisory Committee (SC) of Botany Department, consisting of three senior professors of the department. The methods employed during the present study were designed with the sole purpose of seeking / eliciting the precious wealth of information on the ethno-veterinary uses of medicinal plants practiced by the natives of the Kashmir Himalaya. Field surveys were conducted in various localities inhabited by the local communities during different seasons of the year 2012-15. Some of these localities are: Surgan, Kalay Pani, Bukwali, Kel, Arangkel, Domail Bala, Shounther, Lunda Nar, Janawaii, Phulawaii, Halmat, Taobutt etc., Usually, the elderly and experienced members of the tribes, locally known as `Budhair' (aged) preferably above the age of forty, were interviewed. More often, they were accompanied to the field for the identification of plant species used and their preferred habitats. The survey targeted farmers, shepherds, pastoralists, traditional healers, gardeners, shopkeepers and plant collectors who have the knowledge of veterinary practices. The plant specimens collected from the alpine and subalpine pastures were shown to them for authentication. All the relevant information, in particular the mode of preparation, method of use and dosage of each medicinal plant species was recorded. To bring an element of accuracy, the information obtained from one locality was cross-checked with that of others. Distribution status of the plant species used in the veterinary practices in the region (critically endangered, endangered, vulnerable and secure) was also determined on the basis of field observation and information collected from the inhabitants of the area.

**Plant identification and herbarium deposition**

Specimens of medicinal plants collected from each locality were provided with a collection number for future reference and supported by check lists for inventory. The plant specimens collected were processed at the Herbarium of Botany Department, University of Azad Jammu & Kashmir, Muzaffarabad and then identified with the help of available literature [36-38]. The properly processed plant specimens have been deposited in the Herbarium of Botany Department, University of Azad Jammu & Kashmir, Muzaffarabad.

**Data Analysis**

***Relative frequency citation (RFC)***

Relative frequency of citation was calculated using RFC=FC/N

Where FC =is the number of informants reporting the use of plant divided by the sum of informants who took part in the study (N) [7].

While, RFC = number of citation (for a given species) divided by number of citations for all species [39].

The frequency of citation was calculated to assess the incidence of one particular plant species used for the treatment of veterinary diseases in relation to the overall citations for all plants. The frequency of citation for a plant species was calculated as follows: Frequency of citation for a particular species = (Number of citations for that particular species/Number of all citations for all species)*100.

***Use Value (UV)***

The relative importance of each species was computed according to the given formula:

$UVs =\sum\frac{UVi}{No},$ proposed by Phillips and Gentry (1993) [66]; Where ‘UVi” represents use value for a given species among the informants who participated, and ‘Ni’ represents the sum of informants.

**Results**

**Demography and data collection**: For the collection and documentation of demographic information well informed persons of the relevant area were approached for interviews and group discussion in accordance with the standardized questionnaires furnished for this purpose. In order to collect, the ethnoveterinary information, the data was gathered from the informants, conducting extensive field visits during the year 2012-2015 going through pre-planned questionnaires using standardized data collecting protocols [40-42]. A total of 126 informants were either interviewed at their homes, in the field or at the religious places through convenience sampling. Among these, 73 were the females and 53 were the male, young informants (43) were between the ages of 30-45 years, 56 were of the age 40-60 years and sixteen were 61 to 75 years old. Rest of the 11 informants were of the age of 76 or above (Table 1). Majority of the informants (87) were illiterates and 26 informants were having 10 to 12 years of education while 13 informants were holding graduation level degrees. During interviews, it was observed that the illiterate and old age group informants have more traditional knowledge of plants than young and educated class. Females of above 40 years of age were found more informative and true practitioner of the ethnoveterinary sector. All the informants were interviewed in local language Pahari/Hindko/Kashmiri. The key questions on Ethnoveterinary were on local names of plants and their parts used, mode of preparation and administration, amount of dose given, disease treated and personal experience of informants. Code of ethics of International Society of Ethnobiology (2008) was followed during data collection (<http://ethnobiology.net/code-of-ethics/>). As the data collection was about the animals, therefore, the people who were in close interaction with the cattle/animals were targeted. After complete briefings to the informants about the purpose of this research work, verbal consents were taken from all the localities from where the data has been collected. As most of the informants were illiterate and it was not possible to take written consent from them.

**Table 1. Informant’s demographics in the study area.**

| **Gender** | **Education Level** | **Occupation** | **No. of informants** |
| --- | --- | --- | --- |
| **Female** | Illiterate | Healer | 16 |
|  |  | Plant collector | 27 |
|  |  | Shepherd | 14 |
|  | Illiterate total | | 57 |
|  | Matric / Intermediate | Plant collector | 7 |
|  |  | Shepherd | 4 |
|  | Matric total | | 11 |
|  | Graduation | Plant collector | 3 |
|  |  | Shepherd | 2 |
|  | Graduation total | | 5 |
| **Female total** | | | **73** |
| **Male** | Illiterate | Elder Non-professional | 7 |
|  |  | Farmer | 8 |

|  |  | Gardener | 2 |
| --- | --- | --- | --- |
|  |  | Healers | 3 |
|  |  | Plant collector | 6 |
|  |  | Shepherd | 2 |
|  |  | Healer | 2 |
|  | Illiterate total | | 30 |
|  | Matric/Intermediate | Elder Non-professional | 3 |
|  |  | Farmer | 2 |
|  |  | Gardener | 1 |
|  |  | Healer | 1 |
|  |  | Plant collector | 4 |
|  |  | Shepherd | 1 |
|  |  | Shopkeeper | 2 |
|  |  | Trader | 1 |
|  | Matric/Intermediate total | | 15 |
|  | Intermediate total | |  |
|  | Graduation | Elder Non-professional | 1 |
|  |  | Farmer | 1 |
|  |  | Gardener | 1 |
|  |  | Healer | 1 |
|  |  | Plant collector | 2 |
|  |  | Shepherd | 1 |
|  |  | Shopkeeper | 1 |
|  | Graduation Total | | 8 |
| **Male total** | | | **53** |
| **Grand total** | | | **126** |

***Taxonomic distribution and growth form of medicinal plants***

The current study reported 39 medicinal plants belonging to 21 families, which were used for the treatment of 21 livestock diseases (Table 2). Polygonaceae was the dominant family with 7 species; followed by Crassulaceae (5 species); Asteraceae (4 species), Papilionaceae (3 species) and Lamiaceae, Apiaceae, Caprifoliaceae (2 species each). The remaining 11 families were represented by one species each (Fig.2).

***Plant part used, formulation and use categories***

The informations regarding the usage of parts of the plants were obtained from the participants revealed that different parts of the plants are used for preparation of remedies. Roots were the most used parts (49%) followed by aerial parts (28%), seeds (8%), fruits (8%), barks and resins are used (2%) each, while leaves with (3%) in the veterinary treatments. The main method for preparation of the remedies was mashed uncooked (18 species), Cooked (08 species), decoction (03 species) and powder and resin (one species each). The key informants in this study reported 21 major therapeutic uses of the plants which included Enterotoxaemia, Dysentery, Indigestion, Internal heat, Dehydration, Tonic, Milk production, Ecto-parasitism, Post-delivery treatment, Anti-salt, Hemoglobinuria, Prolapse of uterus, PPR, Dyspnea, Repeat breeding, Goat pox, Deworming, Nephritis, Strangles, Constipation and Cough (Table 2, Fig. 3). A total of 9 species were used as Tonic, 9 in indigestion, 4 species for Post-delivery treatment, deworming and constipations each, 03 for Dysentery, 02 for each Enterotoxaemia, dyspnea, internal heat, milk production, cough, goat pox, one for Anti-salt, dehydration, repeat breeding, nephritis, PPR, strangles, hemoglobinuria and ecto-parasitism.

Medicinal plants used as tonic are *Saussurea lappa, Aralia cachemiriana, Bistorta amplexicaulis, B. affinis, Helianthus annus, Geranium wallichianum, Berberis lycium, Aesculus indica and Angelica cyclocarpa*. Plant species used for the treatment of indigestion include: *Aesculus indica, Thymus linearis, Saussurea lappa, Angelica archangelica, A. cyclocarpa, Rumex nepalensis, Zea mays* and *Viburnum grandiflorum.* Plant species used to cure post-delivery treatments include: *Dipsacus inermis, Rumex acetosa, Rumex nepalensis* and *Taraxacum laevigatum.*

**Figure 3: Plant Parts used to cure different disease in the animals**

Each plant species is provided with its scientific name and author citation; followed by the family to which it belongs to; local name (in italics); growth form; altitudinal range in meters above mean sea level); distribution status in the region (critically endangered, endangered, vulnerable and secure); and lastly in brief the part (s) used and the mode of preparation and the dosage (wherever available). The sequence of the plant species in an alphabetical order is as follows.

**Figure 2: Family-wise distribution of the plants used for veterinary treatments**

**Figure 3: Frequency of the Plant Species used against different disease categories**

**Figure 4: Proportion of life form of the plant species used in ethnoveterinary**

**Table 2. Ethno-Veterinary uses of the plants of Sharda Division, Neelum Valley AJK.**

| **S. No.** | **Plant name** | **Family** | **Local name** | **Habit** | **Alt. Range (m)** | **Current Status** | **Part Used** | **Ethno-veterinary uses** |
| --- | --- | --- | --- | --- | --- | --- | --- | --- |
| 1 | *Aconogonon molle* (D.Don) H. Hara | Polygonaceae | *Chukro* | Herb | 2000-3000 | *Secure* | Rt | Mashed uncooked roots are given orally to cure Enterotoxaemia problems (*Andran Da Taap*). Roots after boiling in the water, along with molasses, in solution form are fed orally to cure lamb dysentery (PPR). |
| 2 | *Aconogonon rumicifolium* (Royle ex Rab.) H.Hara | Polygonaceae | *Panchoola* | Herb | 2500-3500 | *Secure* | Rt | Mashed uncooked roots are given orally to cure Enterotoxaemia problems. |
| 3 | *Aesculus indica* (Wall. ex Camb.) Hook.f. | Hippocastanaceae | *Bunkhoor* | Tree | 2000-2800 | *Secure* | Frt | Fruits are mashed and fed to the cattle to treat indigestion *(Malla)* as it has warm effect. Seeds are also given orally as tonic, especially to the horses. |
| 4 | *Ajuga bracteosa* Wall. ex Benth. | Lamiaceae | *Jan e Adam* | Herb | 2500-3500 | *Vulnerable* | Rt | Uncooked roots are given orally to the cattle suffering from internal heat (*Peelia*). |
| 5 | *Angelica archangelica var. himalica* (Clarke) E.Nasir | Apiaceae | *Murchar* | Shrub | 2000-3000 | *Secure* | Rt | Roots of the plants are cooked and with the addition of molasses are given to cure indigestion cause by the cold. It increases internal temperature and relieve the pain. It is also used to cure Dyspnea *(Dhansna).* |
| 6 | *Angelica cyclocarpa* (Norman) Cannon | Apiaceae | *Chora* | Shrub | 2000-3500 | *Vulnerable* | Rt | Indigestion (locally known as *Dood da Mala*) in cattle is cured by giving uncooked roots with the addition of molasses. Same roots, while cooked are given to the cattle to cure indigestion caused by the cold (locally known as *Thanady da malla*). Used to cure animal's dehydration (*Taku*) issue which usually results because of the non-availability of the water for a long time. |
| 7 | *Aralia cachemirica* Dcne. | Araliaceae | *Chooryal* | Shrub | 1800-2500 | *Secure* | Rt | Mashed and uncooked roots are given to the cattle as tonic which also increases the production of milk. |
| 8 | *Berberis lycium* Royle | Berberidaceae | *Sunmbal* | Shrub | 1800-2700 | *Secure* | Brk | The bark of the root and stem is peeled off, dried, grinded and then used in combination with rice, maize floor and butter as tonic and to strength the bones and treatment of internal fractures. |
| 9 | *Bistorta amplexicaulis var. amplexicaulis* | Polygonaceae | *Chiti Masloon* | Herb | 2000-3500 | *Secure* | Rt | Cooked roots (decoction) are given to the feeble cattle as tonic. |
| 10 | *Bistorta amplexicaulis var. speciosa* (Meisn.) Munshi & Javeid | Polygonaceae | *Bari Masloon* | Herb | 1800-2500 | *Secure* | Rt | Cooked roots are given to the feeble cattle as tonic. |
| 11 | *Capsicum annuum* L. | Solanaceae | *Rattian Marchan* | Shrub | 1800-2000 | *Cultivated* | Frt | Cotton cloth kept on hay and burnt. Dried fruits of *Capsicum annuum* (locally called *Rattian marchan*) are grinded and the powder in combination with sugar is also poured on the fire. Smoke and fumes produced are being forcibly inhaled to animal (horse/mule/donkey). Consequently, there is copious discharge from nasal cavities and animals become healthy. The disease is known as Strangles (locally called *Kannar*). |
| 12 | *Cedrus deodara* (Roxb. ex D.Don) G.Don | Pinaceae | *Pluddar* | Tree | 1800-2250 | *Vulnerable* | Rsn | Resin extracted from the trunk of *Cedrus deodara* after heating the chopped parts of the woods, is applied on the affected skin to cure/mange ecto-parasitism. Burned mobile oil is also used for the same purpose |
| 13 | Curcuma longa L. | Zingiberaceae | *Liddhar* | Herb | 1800-2300 | *Cultivated Secure* | Rt | Roots of *Curcuma longa* (Haldi) are cooked in ghee are also fed orally to cure the issue of Prolapse of Uterus. |
| 14 | *Dipsacus inermis* Wall. ex Roxb. | Dipsaceae | *Pilha* | Herb | 1800-2500 | *Secure* | Rt | About 1-2 kg of roots are mashed and cooked in water and given orally to expel placenta as post-delivery treatment in cattle. This has shown quick results and placenta is removed.  Cooked Roots of *Dipsacus inermis* (Pilha) are also used to cure Prolapse of Uterus (*Mongra Ana / Bhar Ana)*. |
| 15 | *Geranium wallichianum* D.Don. ex Sweet | Geraniaceae | *Ratanjoog* | Herb | 1800-3000 | *Secure* | Rt | Cooked roots are given to the cattle as tonic. |
| 16 | *Helianthus annus* L. | Asteraceae | *Gul e Aftab* | Shrub | 1800-2300 | *Cultivated Secure* | Sd | Crushed seed (Powder) are given to the week cattle orally as tonic to the general weakness. |
| 17 | *Hylotelephium ewersii* (Ledeb.)H. Ohba | Crassulaceae | *Loonslooni* | Herb | 2500-3500 | *Secure* | AP | Whole mashed uncooked plant is fed to the goats and sheep to reduce the effects of over dozed salts, hence known as *Loonslooni* (*Loon* is local name of Salt). |
| 18 | *Indigofera heterantha var. heterantha* | Papilionaceae | *Kainthi* | Shrub | 1800-2800 | *Secure* | Rt | Mashed uncooked roots are given to the young cattle as dewormer. |
| 19 | *Lavatera cachemiriana var. cachemiriana* S.Abdin | Malvaceae | *Dang Sonchal* | Shrub | 2000-2800 | *Vulnerable* | Rt | Cooked roots are used to treat constipation in the animals. |
| 20 | *Ligularia amplexicaulis* DC. | Asteraceae | *Mata Khaish* | Herb | 2800-3600 | *Secure* | Rt | Crushed roots are given orally uncooked to the young cattles to expel worms from the abdomen. It increase digestion and helps the young ones to graze fresh grass. |
| 21 | *Phaseolus lunatus* Linn. | Papilionaceae | *Mooth* | Climber | 1800-2400 | *Cultivated Secure* | Sd | Seeds (Mooth*)* after boiling in the water are fed, so that blister should appear on the outer surface of the animal to cure Goat Pox (*Thandian*). Otherwise, the death of the animal is possible. |
| 22 | *Phaseolus vulgaris* Linn. | Papilionaceae | *Mooth* | Climber | 1800-2400 | *Cultivated Secure* | Sd | Seeds (Moth*)* after boiling in the water are fed, so that blister should appear on the outer surface of the animal to cure Goat Pox (*Thandian*). Otherwise, the death of the animal is possible. |
| 23 | *Punica granatum* L | Lythraceae | *Darru* | Shrub/tree | - | *Cultivated* | Frt | Other than the *Alum (Phatkri)*, outer fleshy part of the fruit of *Punica granatum* L. (locally known as *Darru*) is dried, grinded and mixed in yogurt and fed orally to cure the issue of nephritis locally called *Dkahotra/ Chulkna*. |
| 24 | *Rheum webbianum* Royle | Polygonaceae | *Chootyal* | Herb | 2500-3600 | *Vulnerable* | Rt | Mashed roots are given to cure indigestion and constipation issues in cattles. Mashed roots are also tied on the external injuries to relieve pain in the cattles. |
| 25 | *Rhodiola himalensis* (D.Don.)S.H.Fu. | Crassulaceae | *Bugomasti* | Herb | 2700-3600 | *Secure* | AP | Aerial parts are crushed and fed to the young cattle are dewormer. |
| 26 | *Rhodiola pinnatifida* Boiss. | Crassulaceae | *Bugomasti* | Herb | 2800-3600 | *Secure* | AP | Aerial parts are crushed and given to the young cattle are dewormer |
| 27 | *Rhodiola sp.* 1 | Crassulaceae | *Bugomasti* | Herb | 2600-3500 | *Secure* | AP | Aerial parts are crushed and given to the young cattle are dewormer |
| 28 | *Rumex acetosa* L. | Polygonaceae | *Sufaid Hoola* | Herb | 1800-3000 | *Secure* | Rt | Cooked roots are believed effective to cure cough, Indigestion and constipation. Roots are buried under the fire in ash and used to expel retained placenta as a post-delivery complication and also on cough. |
| 29 | *Rumex nepalensis* Spreng. | Polygonaceae | *Hoola* | Herb | 1800-3000 | *Secure* | Rt | Cooked roots are believed effective to cure cough, Indigestion and constipation. Roots are buried under the fire in ash and used to expel retained placenta as a post-delivery complication and also on cough. It is also used to cure Dyspnea *(Dhansna).* |
| 30 | *Saussurea lappa* (Dcne.)Sch. Costus (Falc. Lipsch.) | Asteraceae | *Kuth* | Herb | 2500-3500 | *Critically Endangered* | Rt | Crushed roots are given uncooked to the sheep and goats to expel worms and also believed as tonic. The cattle start eating after the treatment. |
| 31 | *Sedum trullipetalum* H&T. | Crassulaceae | *Loonslooni* | Herb | 2500-3600 | *Secure* | AP | Un cooked, mashed whole plant is given to the goats and sheep to reduce the effects of over dozed salts, hence known as *Loon slooni* (*Loon* is local name of Salt). |
| 32 | *Taraxacum laevigatum* (Willd.)DC | Asteraceae | *Hand* | Herb | 1800-3200 | *Secure* | Rt | Mashed uncooked roots are given to cure the post-delivery complication especially to expel retained placenta in the cattle. |
| 33 | Trigonella foenum-graecum Linn. | Fabaceae | *Sinji* | Herb | 1800-2600 | *Cultivated Secure* | AP | *Trigonella foenum-graecum* (Maithi) is boiled and fed orally to the animals for the purpose to cure Prolapse of Uterus. |
| 34 | *Thymus linearis* Benth. | Lamiaceae | *Ajwain/ Bun jamain* | Herb | 2800-3500 | *Endangered* | AP | Decoction of the whole plant with addition of milk, maize flour and molasses is orally fed to the animals suffering from Indigestion (*Malla*) and Hemoglobinuria (*Rut Mortrna*). |
| 35 | *Urtica dioica* L. | Urticaceae | *Kairi* | Herb | 1800-2800 | *Secure* | Lvs | Vagical palpation with irritation, causing plant *Urtica dioica* (Kari), leaves is practiced which cause irritation in the birth canal and eventually animal is set into heat cycle called Repeat Breeding (*Na Thairna*). |
| 36 | *Verbascum thapsus* L. | Saxifragaceae | *Gadikan* | Herb | 1800-3000 | *Secure* | AP | Leaves are cooked and given to the cattle to relieve pain in case of injury. Broad leaves are also lapped on the injured parts to relieve pain. |
| 37 | *Viburnum cotinifolium* D.Don. | Caprifoliaceae | *Ukloon/ Guch* | Shrub | 1800-2800 | *Secure* | AP | Tips of the plants are collected while starting sprouting and are given uncooked orally to the horses and buffalos to cure constipations. |
| 38 | *Viburnum grandiflorum* Wall. ex DC. | Caprifoliaceae | *Ukloon/*  *Guch* | Herb | 1800-2800 | *Secure* | AP | Sprouting tips of the plants are collected mashed and given to the horses and buffalos uncooked to cure constipations. |
| 39 | *Zea mays* L. | Poaceae | *Makai* | Herb | 1800-2500 | *Cultivated* | AP | Young plants of *Maize* (Makai) dried under shad, are boiled and the hot plant parts are tied on the back of animals (cows & buffalos) which is believed to dry the internal fluid from the body of the animal, which is another type of “indigestion” (locally known as *Linga da Malla*) |

Frt = Fruit, Lvs = Leaves, Rt = Root, AP = Aerial Parts, WP = Whole Plant, Sd = Seeds, Flr = Flowers, Rsn = Resin,

**Relative Frequency of Citation and Use Value**

Relative Frequency of Citation (RFC) and Use Value (UV) of the medicinal plants was calculated ranging between (41) to (7.32) (Table 5 Relative Frequency of Citation and Use Value). The highest Relative Frequency Citation was found for *Saussurea lappa* (41/7.32), followed by *Rumex acetosa* (37/6.61), *Rumex nepalensis* (36/6.43), *Thymus linearis* (28/5.0) and *Angelica cyclocarpa* (28/5.0). The lowest relative frequency of citation was recorded by *Rhodiola pinnatifida, Taraxacum laevigatum* and *Helianthus annus* (5/0.89 each). The highest use value was recorded for *Saussurea lappa* (0.33), followed by *Rumex acetosa* (0.29), *Rumex nepalensis* (0.29), *Thymus linearis* and *Angelica cyclocarpa* (0.22 each) (Table 02 Diseases and plants used to cure the ailments). The lowest use value was recorded in *Rhodiola pinnatifida* and Taraxacum laevigatum, which was 0.04 each.

**Table 3. Relative Frequency of Citation and Use Value**

| **S. No.** | **Plant name** | **FC** | **UV** | **RFC** |
| --- | --- | --- | --- | --- |
| 1 | *Aconogonon molle* | 25 | 0.20 | 4.46 |
| 2 | *Aconogonon rumicifolium* | 15 | 0.12 | 2.68 |
| 3 | *Aesculus indica* | 19 | 0.15 | 3.39 |
| 4 | *Ajuga bracteosa* | 13 | 0.10 | 2.32 |
| 5 | *Angelica archangelica var. himalica* | 20 | 0.16 | 3.57 |
| 6 | *Angelica cyclocarpa* | 28 | 0.22 | 5.00 |
| 7 | *Aralia cachemirica* | 18 | 0.14 | 3.21 |
| 8 | *Berberis lycium* | 16 | 0.13 | 2.86 |
| 9 | *Bistorta amplexicaulis* | 12 | 0.10 | 2.14 |
| 10 | *Bistorta amplexicaulis var. speciosa* | 13 | 0.10 | 2.32 |
| 11 | *Capsicum annuum* | 9 | 0.07 | 1.61 |
| 12 | *Cedrus deodara* | 9 | 0.07 | 1.61 |
| 13 | *Curcuma longa* | 7 | 0.06 | 1.25 |
| 14 | *Dipsacus inermis* | 27 | 0.21 | 4.82 |
| 15 | *Geranium wallichianum* | 14 | 0.11 | 2.50 |
| 16 | *Helianthus annus* | 5 | 0.04 | 0.89 |
| 17 | *Hylotelephium ewersii* | 6 | 0.05 | 1.07 |
| 18 | *Indigofera heterantha var. heterantha* | 7 | 0.06 | 1.25 |
| 19 | *Lavatera cachemiriana var. cachemiriana* | 11 | 0.09 | 1.96 |
| 20 | *Ligularia amplexicaulis* DC. | 18 | 0.14 | 3.21 |
| 21 | *Phaseolus lunatus* | 6 | 0.05 | 1.07 |
| 22 | *Phaseolus vulgaris* | 7 | 0.06 | 1.25 |
| 23 | *Punica granatum* | 6 | 0.05 | 1.07 |
| 24 | *Rheum webbianum* | 12 | 0.10 | 2.14 |
| 25 | *Rhodiola himalensis* | 6 | 0.05 | 1.07 |
| 26 | *Rhodiola pinnatifida* | 5 | 0.04 | 0.89 |
| 27 | *Rhodiola sp.* 1 | 6 | 0.05 | 1.07 |
| 28 | *Rumex acetosa* | 37 | 0.29 | 6.61 |
| 29 | *Rumex nepalensis* | 36 | 0.29 | 6.43 |
| 30 | *Saussurea lappa* | 41 | 0.33 | 7.32 |
| 31 | *Sedum trullipetalum* | 6 | 0.05 | 1.07 |
| 32 | *Taraxacum laevigatum* | 5 | 0.04 | 0.89 |
| 33 | *Trigonella foenum*-*graecum* | 7 | 0.06 | 1.25 |
| 34 | *Thymus linearis* | 28 | 0.22 | 5.00 |
| 35 | *Urtica dioica* | 17 | 0.13 | 3.04 |
| 36 | *Verbascum thapsus* | 7 | 0.06 | 1.25 |
| 37 | *Viburnum cotinifolium* | 14 | 0.11 | 2.50 |
| 38 | *Viburnum grandiflorum* | 15 | 0.12 | 2.68 |
| 39 | *Zea mays* | 7 | 0.06 | 1.25 |

**DISCUSSION**

By the discovery of the new drugs and a virtuous use of traditional medicine, it is very essential to record and preserve the traditional knowledge on medicinal plants those intended for treating humans and animals. Furthermore, the botanical knowledge is instrumental for the correct identification of the plant species, hence very important for avoiding errors in the gathering of medicinal plants. The present study has reported the ethno-veterinary medicinal uses of 39 angiosperm and gymnosperm plant species used by the local inhabitants and nomads of the Neelum Valley, Kashmir Himalaya. The respondents involved in the study through field interactions were 73 females and 53 males. Females from the study area were focused because of their close interaction with the animals as compared to the males. The results show that people above 50 years of age had more knowledge about the traditional use of the medicinal plants to cure various ailments of the domestic animals [43]. These medicinal plant species belong to 31 genera and 21 families and majority of them are perennial herbs. The most species rich family was Polygonaceae followed by the Asteraceae and Crassulaceae. Different plant parts, such as leaves, fruit, seeds, roots, etc. are used. The wide use of identic common names indicates a broad transmission of indigenous knowledge of Ethnoveterinary plants holding different uses [44]. The routes of administration of these herbal remedies were essentially oral, followed by the topical application. The part used was mostly the roots of the plants followed by the aerial part as a whole or the leaves. During the present study, the leading growth form of the medicinal species was herbs (62%), shrubs (25%), and trees (8%) indicated in Table 4. Herbs are often used because of their frequent availability, ease of collection and applications [45-46]. Plant species are distributed along wide altitudinal range starting from 1800 up to 3700 m (amsl). They grow in diverse range of habitats, such as valley plains, montane forests, subalpine and alpine pastures. In general, these plant species through different modes of preparation to form crude drugs are fed as food supplements to promote faster weight gain, as enterotoxaemia, indigestion, dehydration, ecto-parasitism, post-delivery complications, dewormer, relieve constipation, respiratory and reproductive disorders [47]. The relative high frequency of some plants may allow the researchers in related academic disciplines for future drug discovery [48]. These plant species should be focused for the conservation, as their dynamic uses may cause threat to their population by over harvesting. *Sedum* stem is used to heal wounds and relieve pain in cattle while *Rumex acetosa* root powder in combination with *Taraxacum officinale* and curd is given to the cattle suffering from liver-fluke and digestive disorder in the Western Himalaya. Similarly, *Rumex nepalensis* roots are used in combination with milk of the cow and salt to cure the juvenile infection of the newly born calf till the age of one month in the Himalaya [49]. *Geranium wallichianum* rhizome is used to treat dislocation of the locomotion organs, inflammation of hooves, warts and abscissions*. Angelica glauca* rhizomes are used to enhance milk production in cattle, particularly in cows and goats, in the Western Himalaya [49]. *Verbascum thapsus* aerial parts are used to cure inflammation in cows, buffalos and sheep in the Himalaya [49]. It is also practiced against rabies as it causes vomiting [50]. These plants have been reported from the study area but having different medicinal values.

*Taraxacum officinale* is used in combination with *Acacia modesta* (Phlai) to enhance milk production while *Aesculus indica* nuts are colic and used to cure chest diseases of horses, donkeys and mules and used as stimulant in the Poonch division of Kashmir Himalaya [19]. *Curcuma longa* is used for prevention of miscarriage in the cattle in Kashmir Himalaya [50]. Some herbal preparations used for livestock were made by a combination of two or more plants species or by the addition of some other items like ghee and sugar, which reduce the relative potency of the remedy [29]. The method of administering ethno-veterinary plant remedies varied among different ethnic communities [51, 52].

Traditional uses of the biodiversity itself are complementary phenomena essential for the economic development of the society [53]. Biodiversity of any area and traditional knowledge reflects many generation of experience and problem-solving by the ethnic tribes. It represents an enormously valuable database that provides the baseline information for the commercial exploitation of biological resources [65, 54]. This important information could be useful for the vets, pharmacologists, botanists, etc. interested in the development of alternative therapies. Although, ethnobotanical contributions have been made at country level [56-63] but no detail ethnobotanical survey has been carried out yet in Neelum Valley, Azad Jammu & Kashmir. Therefore, this Ethnoveterinary research was important to record the folklore informations about the local medicinal plants used for the veterinary purpose [64-66]. Biopiracy is the unfair exploitation and monopolization of public domain knowledge and biological resources. In this context, the need of the hour is to speedily document evidence-based reliable information about the biodiversity and its' different uses by the ethnic tribes. Hopefully, the information thus generated, as in the present research, could supplement our efforts in the direction of the local biodiversity registers-a key instrument for achieving the regional and global biodiversity and the conservation goals.

**Conclusion**

Indigenous communities at Neelum Valley are dependent on medicinal plants for Ethno veterinary practices. The people practiced (39) medicinal plants to cure (21) livestock diseases. Knowledge about the traditional medicinal system is restricted to the herders, farmers and elder community member. The younger generation is unaware of this traditional treasure and takes no interest due to modernization. The current study has an important contribution towards the preservation of indigenous plants-based knowledge from extinct. There are several medicinal plants which are being used in traditional herbal system of veterinary disorders. Some of the important are *Dipsacus inermis, Rumex nepalensis*, *Angelica cyclocarpa, Saussurea lappa, Aesculus indica etc.* New Ethnoveterinary uses used at the study area were found for *enterotoxaemia, dehydration, indigestion, dewormer etc*. Polygonaceae is utmost plant family being in use for various livestock ailments. It is suggested that phytochemical and pharmacological investigations must be carried out to isolate the active compound and testing the in vitro or in vivo efficiency of the abovementioned plants against the targeted veterinary diseases. Furthermore, critical toxicological investigations are also required to ensure the safe and secure use of documented ethno-medicines. In order to share and further maintain this knowledge, it is direly needed to aware the rural population about the significance of traditional ethnoveterinary knowledge and to motivate them on the conservation of the natural flora.

**Supporting Information:**

S1: File of Sample of Questionnaire used during field survey for obtaining ethnobotanical information. (DOX)

Figures 1-5

**Acknowledgement:** The authors would like to thank the interviewees and other inhabitants of Neelum valley, AJK, for their contribution to this work.

**Author Contributions**

**Conceptualization**: Sardar Muhammad Rafique Khan, Tanveer Akhter

**Data curation:** Sardar Muhammad Rafique Khan, Mumtaz Hussain

**Formal Analysis:** Sardar Muhammad Rafique Khan, Mumtaz Hussain

**Investigation:** Sardar Muhammad Rafique Khan, Mumtaz Hussain, Tanveer Akhter

**Methodology:** Sardar Muhammad Rafique Khan, Mumtaz Hussain

**Project Administration:** Sardar Muhammad Rafique Khan

**Supervision:** Tanveer Akhter

**Writing-original draft**: Sardar Muhammad Rafique Khan

**Writing-review & editing:** Sardar Muhammad Rafique Khan, Tanveer Akhter

**REFERENCES**

1. Sullivan, K, Shealy C.N. Complete Natural Home Remedies. Element Books Limited, Shaftesbury, UK., 1997; pp: 3-5.
2. Ayeni E.A, Basiri B. Ethnoveterinary survey of plants used in treating livestock among the Fulani people of Girei, Adamawa State, Nigeria. World News of Natural Sciences. 2018; 16: 53-66.
3. Husain, M. Geography of Jammu and Kashmir. Rajesh Publications, New Delhi, India. 2001; pp: 15-16.
4. Dar, M.E. Ethnobotanical Uses of Plants of Lawat District Muzaffarabad, Azad Jammu and Kashmir Asian J. Plant Sci., 2003; 2: 680-682.
5. Lawrence, W.R. The Valley of Kashmir 1895. (Reprinted). Chinar Publishing House, Srinagar, 1992.
6. Hoareau, L, E.J. Da-Silva. Medicinal plants: A re-emerging health aid. Elect. J. Biotechnol. 1999;Vol 2.
7. Ullah M, Khan MU, Mahmood A, Malik RN, Hussain M, Wazir SM, Daud M, Shinwari ZK. An ethnobotanical survey of indigenous medicinal plants in Wana district, South Waziristan Agency, Pakistan. Journal of Ethno-pharmacology. 2013; 150(3): 918-924.
8. Bullitta S. GA Re, Manunta M.D.I., Piluzza G. Traditional knowledge about plant, animal and mineral based remedies to treat cattle, pigs, horses and other domestic animals in Mediterrianian island of Sardinia. Journal of Ethnobiology and Ethnomedicines. 2018; 14(1): 50.
9. Lans C, Turner N, Khan T, Brauer G, Boepple W. Ethnoveterinary medicines used for ruminants in British Columbia, Canada. Journal of Ethnobiology and Ethnomedicines. 2007; 3(1): 11.
10. Dar, G.H., Vir J, Kachroo P, Buth HH. Ethnobotany of Kashmir 1, Sindh Valley. J. Econ. Tax. Bot,. 1984; 3: 668-675.
11. Kachroo P, Nahvi IM. Ethnobotany of Kashmiris. In: Forest Flora of Srinagar and Plants of Neighbourhood. Singh, G. and P. Kachroo (Eds.), Bishen Singh Mahendra Pal Singh, Dehra Dun, India1987.
12. Ara S, Naqshi AR. Ethnobotanical studies in the Guraise Valley. J. Econ. Tax. Bot., 1992; 17: 657-678.
13. Kaul MK. Medicinal Plants of Kashmir and Ladakh, Temperate and cold Arid Himalaya. Indus Publishing Co. New Delhi. 1997.
14. Shahzad KR, Malik ZH, Qureshi RA. Phytosociological survey of Samahni valley, Bhimber, Azad Kashmir. Pak. J. Forest., 1999; 49: 91-100.
15. Dastagir G. Medicinal Plants of Mai Dhani Hill, Muzaffarabad, Azad Jammu and Kashmir. Hamdard Medicus. 2001; 1: 29-35.
16. Saghir IA, Awan AA, Mir SM, Khan A, Qureshi SJ, Bano S.. Ethnobotanical studies of Chikar and its Allied Areas of District Muzaffarabad. J. Biol. Sci. 2001; 1: 1165-1170.
17. Gorsi MS, Miraj S. Ethenomedicinal survey of plants of Khanabad Village and its Allied Areas, District Gilgit. Asian J. Plant Sci., 2002; 1: 604-615.
18. Gorsi MS, Shahzad R. Medicinal uses of plants with particular reference to the people of Dhirkot, Azad Jammu and Kashmir. Asian J. Plant Sci. 2002; 1: 222-223.

# 19. Khan MA, Khan MA, Hussain M. Ethno Veterinary Medicinal Uses of Plants of Poonch Valley Azad Kashmir. *Pak. J. Weed Sci. Res.,* 2012.18(4): 495-507.

20. Khan ZS, Khuroo AA, Dar GH. Ethnomedicinal survey of Uri, Kashmir Himalaya. Ind. J. Trad. Knowl. 2004; 3: 351-357.
21. Sudarsanum G, Reddy MB, Nagaraju N. Veterinary crude drugs in Rayalaseema, Andhra Pradesh, India. Int. J. Pharm. 1995; 33(1): 52-60.
22. Ole-Midron JO. The Maasai ethno-diagnostic skill of livestock diseases; A road to traditional bio prospecting. J. Ethnopharmacol. 2003; 84(1): 79-83.
23. Lans C, Brown G. Observations on ethnoveterinary medicines in Trinidad and Tobago. J. Prev. Vet. Med. 1998; 35(2): 125- 142.
24. Jost CC, Shermam DM, Thomson EF, Hesselton RM. Kamala (*Mallotus philippinensis*) fruit is ineffective as an antihelmintic against gastrointestinal nematodes in goats indigenous to Balochistan, Pakistan. Small Ruminant Res. 1996; 20: 147-153.
25. Hammond JA, Fielding D, Bishop SC. Prospects for plant anthelmintics in tropical veterinary medicine. Vet. Res. Commun. 1997; 21: 213-228.
26. Akhtar M S. Anthelmintic evaluation of indigenous plants for veterinary usage. Final Research Report (1983-88). University of Agriculture Faisalabad, Pakistan. 1988.
27. Akhtar MS, Ahmad. Comparative efficacy of *Mallotus philippinensis* fruit (Kamala) or Nilzan drug against gastrointestinal cestodes in Beetal goats**.** Small Ruminants Res. 1992; 8: 121-128.
28. Akhtar MS. Khan MNZI, Latif M. Anthelmintic activity of medicinal plants with particular reference to their use in animals in Indo-Pakistan subcontinents. Small Ruminants Res. 2000; 38: 99-107.
29. Jabbar A, Raza MA, Iqbal Z, Khan N. An inventory of the ethnobotanicals used as anthelmintics in the southern Punjab (Pakistan). *Journal of Ethnopharmacol.*, 2006; 108: 152–154
30. Tabassam SM, Iqbal Z, Jabbar A, Ud-Sindhu Z, Abbas R.Z. Documentation of Ethnoveterinary practices and evaluation of *Azadirachta indica* for treatment of sheep mange. *12^th^ AAAP Animal Science Congress*, Bexco, Busan, Korea, 2006.
31. Farooq Z, Iqbal Z, Mushtaq S, Muhammad G, Iqbal MZ, Arshad M. Ethnoveterinary practices for the treatment of parasitic diseases in livestock in Cholistan desert (Pakistan). *J. Ethnopharmaco.,* 2007; 118: 213–219
32. Dilshad S.M.R., Najeeb-ur-Rehman, Iqbal Z, Muhammad G, Iqbal A, Ahmed N. An inventory of the ethnoveterinary practices for reproductive disorders in cattle and buffaloes, Sargodha district of Pakistan. *J. Ethnopharmacol.,* 2008; 117: 393–402.
33. Abbasi AM, Khan SM, Ahmad M, Khan MA, Quave CL, Pieroni A. Botanical Ethnoveterinary therapies in three districts of Lesser Himalayas of Pakistan. Journal of Ethnobiology and Ethno-medicine, 2013; 9(1): 84.
34. Aziz MA, Khan AH, Adnan M, Ullah H. Traditional uses of medicinal plants used by indigenous communities for veterinary practices at Bajaur Agency, Pakistan. Journal of Ethnobiology and Ethnomedicines, 2018; 14 (1): 11.
35. Anonymous, “*AJK at a glance”*. Published by Planning & Development dept. Govt. of Azad Jammu & Kashmir, 2014.
36. Stewart RR. An Annotated Catalogue of the Vascular Plants of West Pakistan and Kashmir, 1972. Fakhri Press, Karachi, Pakistan.
37. Nasir E, Ali SI.. Flora of Pakistan, No. 1-187. 1970-1987. Pakistan Agricultural Research Council, Islamabad, Univ. Karachi, Pakistan.
38. BSI. Flora of India. Botanical Survey of India, 1996. Kolkatta, India.
39. Ocvirk S, Kistler M, Khan S. Talukder SH, Hauner H. Traditional medicinal plants used for the treatment of diabetes in rural and urban areas of Dhaka, Bangladesh. An ethnobotanical survey. Journal of Ethnobiology Ethno-medicine. 2013; 9-43.
40. Alexiades MN.  Selected Guidelines for Ethnobotanical research: A Field Manual, 1996. The New York Botanical Garden, Bronx, N.Y.
41. Martin GJ. Ethnobotany: A Methods Manual. Earths can Publications Ltd, London. 2004.
42. Heinrich M, Edwards HS, Moerman DE, Leonti M. **Ethnopharmacological ﬁeld studies: a critical assessment of their conceptual basis and methods**. J. Ethnopharmacol, 2009; 124 :1-17.
43. Liu YC, Dao ZL, Yang CY, Liu YT, Long CL. Medicinal plants used by Tibetans in Shangri-la, Yunnan, China. Journal of Ethnobiology and Ethnomedicine, 2009; 5: 15.
44. Hart R, Bussmann R. Trans-Himalayan Transmission, or Convergence? Stauntonia (Lardizabalaceae) as an Ethnoveterinary Medicine. Medicina nei Secoli. 2018; 30(3): 929-948.
45. Marwat SK. Ethnophytomedicines for treatment of various diseases in DI Khan district, Pakistan. Sarhad Journal of Agriculture. 2008; 24(2) : 305-315.
46. Barboza RR, De MS, Souto W, Da J, Mourao S. The use of zootherapeutics in folk veterinary medicine in the district of Cubati, Paraiba State, Brazil. Journal. Of Ethnobiology and Ethnomedicine. 2007; 3(1): 32.
47. Leeflang P. Some observations on Ethnoveterinary Medicine in Northern Nigeria,1993.
48. Khattak NS, Nouroz F, Rahman IU, Noreen S. Ethnoveterinary uses of medicinal plants of district Karak, Pakistan. Journal of Ethnopharmacology, 2015; 171: 273-279.
49. Khuroo AA, Malik AH, Dar AR, Dar GH, Khan ZS. Ethno-veterinary medicinal uses of some plant species used by the Gujjar Tribes of Kashmir Himalaya. Asian. J. of Plant Sci. 2007; 6(1): 148-152.
50. Bhatti RC, Nirmala C, Kaur A, Singh S, Kumar P, Kaur R, Sigh AN. Harnessing of local plant species by indigenous people of Hamirpur District for Ethno-veterinary purposes. Annals of Plant Sci. 2017; 6.12: 1898-1925.
51. Eswaran S, Boomibalagan P, Rathinavel S. Ethnoveterinary medicinal practices of the villagers of Usilampatti Taluk of Madurai district, India. International Journal of Botany. 2013; 9: 37-43.
52. Bharati KA. B.L. Sharma. Plants used as Ethnoveterinary medicines in Sikkim Himalayas. Ethnobotany Research and Applications. 2012; 10: 339-356.
53. Dhar U, Rawal RS, Upreti J. Setting priorities for conservation of medicinal plants-a case study in the Indian Himalaya. J. Biol. Conserv. 2000; 95: 57-65.
54. Utkarsh, GM, Gadgil M, Rao PRS. Intellectual property rights on biological resources: Benefiting from biodiversity and peoples knowledge. Curr. Sci., 1999; 77: 1418-1425.
55. Utkarsh, G, Patenting life? Biodiversity and Intellectual Property Rights. Resonance. 2001; 2: 51-56.
56. Ahmad H, Sirajuddin. Ethnobotanical profile of Swat. Proceedings of the 1st Training Workshop on Ethnobotany and its Application to Conservation, September 16-24, 1996, Islamabad, Pakistan. 1996; pp: 202-206.
57. Haq I. Medicinal Plants of Mansehra District. Hamdard Medicus, NWFP, Pakistan, 1993; pp: 63-99.
58. Hocking GM. Pakistan medicinal plants 1. Q. Planta Mater. Vegetation. 1958; 9: 103-119.
59. Hussain F, Khalique A. Ethnobotanical studies on some plants of Dabargia hills, Swat. Proceedings of the 1st Training Workshop on Ethnobotany and its Application to Conservation, (TWEAC'96), NARC, Islamabad. 1996; 207-215.
60. Pie SJ, Manandhar NP. Sources of some local medicines in the Himalayan Regions. Himalayan Ecosyst. 1987; pp: 97-112.
61. Shinwari, ZK, Shah M. The ethnobotany of Kharan district, Baluchistan. Proceedings of the 1st Training Workshop on Ethnobotany and its Applications to Conservation. September 16-25, 1996, NARC, Islamabad. 1996; pp: 124-132.
62. Chaudary MA, Khan MA, Hanif W. Ethno Veterinary Medicinal uses of plants from Samahni Valley, District Bhimber, (Azad Kashmir) Pakistan. Asian J. of Plant Sci. 2006; 5(2): 390-396.
63. Zaman MB, Khan MS. Medicinal plants of West Pakistan. For. Inst. Pak., 1970; 38: 34-46.
64. Croom EM. Documenting and evaluating herbal remedies. Econ. Bot. 1983; 37: 13-27.
65. Dar GH, Bhagat RC, Khan MA. Biodiversity of Kashmir Himalaya, Valley Book House, Srinagar, Kashmir. 2001; pp: 120-122. .

# 66. Phillips OL, Gentry AH. The useful plants of Tambopata, Peru. II: Additional hypothesis testing in quantitative ethnobotany. Economic Botany. 1993; 47: 33-43.

# 67. Majeed M, Bhatti KH, Amjad MS, Abbasi AM, Bussmann RW, Nawaz F, et al. Ethno-veterinary uses of Poaceae in Punjab, Pakistan. PLoS ONE 2020; 15(11): e0241705. https://doi.org/10.1371/journal.pone.0241705
