# Supplementary material for: Ethno-veterinary practice for the treatment of animal diseases in Neelum Valley, Kashmir Himalaya, Pakistan
Source: PLoS One. 2021 Apr 30;16(4):e0250114. doi: 10.1371/journal.pone.0250114 (PMC8087047; doi:10.1371/journal.pone.0250114)
Supplement: Attachment — Submitted filename: Response to Reviewers .docx [file pone.0250114.s007.docx]

**'Response to Reviewers’**

**PONE-D-20-31879**

**ETHNO-VETERINARY PRACTICES USED FOR THE TREATMENT OF ANIMAL DISEASES IN NEELUM VALLEY, KASHMIR HIMALAYA.**

**PLOS ONE**

| **Editors Comments** | | |
| --- | --- | --- |
| **S.No.** | **Editor’s comments** | **Author’s response** |
| 1 | Arrange citation in ascending order | Citations arranged accordingly P. 3, L.43 |
| 2 | Provide a map of the study area with locations. | The study area map has been provided. P. 46, L.119. |
| 3 | Better to adjust in M&M under study area section | Adjusted accordingly. P.3, L.103 |
| 4 | What about the major agriculture crops and dominant plant communities in the study area. | Details are added in the manuscript accordingly.  P. 5, L. 89. |
| 5 | Better to adjust in M&M section under stud area section. | It has been adjusted accordingly.  P. 3, L. 103. |
| 6 | Read following articles | The articles referred were studied and the guidelines were helpful. |
| 7 | Change the figure numbers. | Figure numbers have been revised. |
| **Reviewer-I Comments** (Comments on the manuscript in PDF) | | |
| **S.No.** | **Reviewer’s comments** | **Author’s response** |
| 1 | Ethno-veterinary practices for the treatment of animal diseases in Neelum Valley, Kashmir Himalaya, Pakistan | The title is corrected accordingly.  P.1, L.1 |
| 2 | Rephrase and shorten the abstract. | Needful done accordingly |
| 3 | Please provide justification to the study. | It is further clarified with additions. P.4, L.81-101 |
| 4 | Ethnoveterinary or ethno-veterinary? Be consistent. | It has been corrected for consistency. |
| 5 | This phrase states the key methodology that was carried out. So, please rephrase in short using standard terminology of the methodology that was carried out. | Revised as it was advised. P. 5-7, L. 121-152 |
| 6 | If the region is not the disputed area, then please state the country name. e.g. Azad Jammu & Kashmir, Pakistan or Azad Kashmir, Pakistan?? | Corrected accordingly. P.4, L.84 |
| 7 | What sort of authenticity. What is the significance of quantitative analysis in ethnobotany? Try to link with methods describe in text. | This was basically literature review. The informations collected from the field about uses of the plants were further cross checked from the literature reviewed. |
| 8 | 21 or 29 ailments. Check carefully. | Corrected accordingly. |
| 9 | Please make sure to use species author citation when it appears first time. | It was rechecked and improved.  P. 2. L.25-30 |
| 10 | Some information like first two lines are necessary, otherwise redundant paragraph. | The whole paragraph is important as it refers some important works on ethnobotany. |
| 11 | Ethnobotany is more concerned with people. So, give detail about the focal communities. | Details about the inhabitant communities has been provided already and their dependence on the livestock and agriculture is also discussed. |
| 12 | What does it mean? Make clear. | It mean ‘documentation of the ethnoveterinary knowledge". P. 3, L.60-61 |
| 13 | In fact, this is not only the solution. It would have been more worthy to mention the way we implement for documentation and conservation. | It is clearly mentioned that documentation and conservation are the only solutions, not only documentation……. P.4, L.65-68 |
| 14 | Provide appropriate word. | Improved accordingly. P.4, L.75 |
| 15 | Unclear objective. | It has been revised and improved. P.4, L. 77-79 |
| 16 | Shorten and Rephrase, then move to 4th paragraph. | It has been rephrased and shifted to material & methods section on the recommendations of Editor. P.5-6, L.103-117 |
| 17 | Do not abbreviate at first. | Improved accordingly. P.5, L. 104 |
| 18 | Provide altitudinal range of the study area. | It has been added. P. 5, L. 87-88 |
| 19 | Talk about ethnic groups of communities. | It has been added. Page 5, Line 95-97. |
| 20 | I did not find any standard protocol cited. Is it your own methods or based on literature? | It was already referred on the next page P. 6, L.127. But added here as well. |
| 21 | Have you obtained IRB and PIC before you gather the local people’s knowledge? Make sure you have adopted ethics of ethnobotany. | It has been followed and referred on P. 7. L.147-148 as well. |
| 22 | Clarify, why seasonal documentation/surveys are important. | The study area lies on the high altitude ranging from 2000-6325m. It remain covered with snow for 3-5 months and in the spring season, the local population as well as nomads migrates from the low altitudes to high altitudinal areas where they stay in summer and they return back in the autumn, therefore, data of different seasons was important to get a clear picture of the area. |
| 23 | Be consistency. Either 2012-15 or 2012-2015. | It is 2012-15 consistently. |
| 24 | Have you concerned about poisonous plant identification? This can be serious issue for veterinary purpose in plant identification. | Yes it was a serious concern during the field work and identification process. Being local from the study area, I was aware enough with the culture and customs and therefore, it was easy for me to discuss all the things in detail. Secondly, I have identified all the plants very carefully. |
| 25 | Very limited ethnobotanical indices. | Question/comment is not clear |
|  | Reference? | Reference [7] on the same paragraph P.8, L.160 |
| 26 | Reference?? | Reference [66] in the same paragraph P.9, L.164 |
| 27 | Table???? | Table 1 (Informant’s demographics in the study area) is referred at the end of this paragraph. P.10, L.192 |
| 28 | Appropriate for methods. Think of it. | It has been shifted in Material and Methods section under data collection heading. P.7, L.148 |
| 29 | Unequal ratio of male and female respondent?? Unsatisfactory reason. | The justification is very strong based on field data and cultural realities of area. In the study area, female are solely handling the livestock. In summer season, females are shifted on the pastures with their cattles and are accompanied by the elder males (mostly above 60 years) who have nothing to do in the agriculture fields and other businesses. The young male visits the pastures rarely. Therefore, the females were available with maximum informations. |
| 30 | 39 or 36 medicinal plants. Check carefully. | 39 plant species have been reported from the study area used for the treatment of the livestock. |
| 31 | Not clear legend. Provide legend for two colours. | The graph has been revised accordingly. The legends are also improved. |
| 32 | Figure not provided. | Figure has been added |
| 33 | It should be Figure 1 | It has been changed as Figure 1. P.6, L.119 |
| 34 | IUCN status or else? cite | This is basically conservation status of the plants in the study area. |
| 35 | Citation to the use is essential as far as possible. | These are basically the indigenous uses of the plants which has been documented from the study area. So it has not been mixed with the cited literature. Therefore, citations are not required here. |
| 36 | Check spelling errors for all. | All the spellings have been rechecked. |
| 37 | L or L. or Linn.?? Check author citation for all. Go through Tropics. | It has been rechecked. There is variations in the literature. In author citation, in certain floras, some plants were found with author citation L. while in others it was Linn. for the same author. So, It has been written accordingly. |
| 38 | Table 3 can be appended in Table 2. Otherwise proved full species name with author citation. | As full name of the plant along with citation has been provided in Table 2, therefore, it was avoided in Table 3. List of the plants is same in both tables, therefore, repetition was avoided to save space in the tables. |
| 39 | Is there sp2??? If not just Rhodiola sp. is OK. | It has been corrected as *Rhodiola sp*. S.No. 27 Table 2. |
| 40 | Disseminating medical knowledge should be cautious as some unidentified plants could be poisonous and fatal to animal. So proper taxonomy is essential. Make sure you have stated. | It has already been mentioned under Discussion section. P. 31, L. 272-274 |
| 41 | Have you classified plants by angiosperm and gymnosperms during plant identification? If not do not mention. | It has been revised. The words angiosperms and gymnosperms has been abolished from the discussion section. P. 31, L. 274. |
| 42 | Have you adopted any protocol to select the number of respondent or just a random selection? | It was random selection. |
| 43 | a.s.l | It has been corrected accordingly. P.32, L.290 |
| 44 | This paragraph is informative and seems to be more justification of the study. | It has its importance in the discussion section as well. By this, we can prove that the present research work was important and the ultimate goal has been achieved at least to some extent. |
| 45 | Not clear. What do you want to say. | It has been further clarified with some additions. P. 34, L. 340-343. |
| 46 | Guidelines not followed. | It has been revised as per guidelines. |
| **Reviewer-III Comments** (Comments on the manuscript in PDF) | | |
| **S.No.** | **Reviewer’s comments** | **Author’s response** |
| 1 | Please spell out | Corrected. P. 3, L. 57 |
| 2 | Why it is written in bold? | Corrected P. 4, L. 65 |
| 3 | No need to write both words. | Corrected P.4, L 66 |
| 4 | It seems the whole paragraph is copied either from thesis or internet source; revised them and in the end you need to justify your study with clear objectives; to address which gap has been filled through this study? Why this study is important also mention the ethnoveterinary relevance? | The whole paragraph is self-generated and not copies from any source. It has been revised and improved and shifted in the material and method section. The objective and justification of the study are also revised and improved. Livestock being an important part in the livelihood of the local communities, therefore, the ethno veterinary uses are of prime importance as easier and cheaper solution to cure their cattles. It is also mentioned in the paper. |
| 5 | Your study area need more information e.g the altitudinal variation, rainfall pattern, cattle farms data if available. | It has been improved and informations have been added. P. 4-5, L. 81-100 |
| 6 | No need to write both words. | Corrected P.4, L.81 |
| 7 | No need to write both words | Corrected P.6, L.129 |
| 8 | No need to write both words | Corrected P.6, L.130 |
| 9 | Rewrite the whole sentence to make it clear | It has been improved accordingly  P.7, L.137-138 |
| 10 | How the plant species were identified from these checklists? (An Annotated Catalogue of the Vascular Plants of West Pakistan and Kashmir, and BSI. Flora of India. Botanical Survey of India). | All the cited books and floras are helpful for identification of the plants and for the distribution of the plants in the region. Other than these two, Flora of Pakistan was also consulted and helpful in identification of the plants. |
| 11 | Please correct it demographic | Corrected P.6, L.122 |
| 12 | Ethnoveterinary | Corrected |
| 13 | Please write only animas or cattle no need to write both words | Corrected accordingly P.7, L.149 |
| 14 | Please don't capitalize the therapeutic use in the middle of sentences it should be written in lowercase | Corrected accordingly. P.12-13, L.208-213 |
| 15 | I can see identification issue of plant specimens throughout the manuscript. Please provide clear botanical identity of the plant species cited in the study. I will highlight in the table which need clear identification. | The highlighted plant species are further confirmed from the flora of Pakistan and Tropics. Identification of the plant species was made very carefully by consulting the literature and herbarium of QAU Islamabad and University of AJ&K, Muzaffarabad as well. |
| 16 | provide authority citation | Citation is added |
| 17 | Strangles | Corrected |
| 18 | Roots | Corrected |
| 19 | Goat pox | Corrected |
| 20 | Indigestion | Corrected |
|  |  |  |
| **General requirements** | | |
| 1 | Please ensure that your manuscript meets PLOS ONE's style requirements, including those for file naming. The PLOS ONE style templates can be found at <https://journals.plos.org/plosone/s/file?id=wjVg/PLOSOne_formatting_sample_main_body.pdf> and <https://journals.plos.org/plosone/s/file?id=ba62/PLOSOne_formatting_sample_title_authors_affiliations.pdf> | I have formatted my manuscript accordingly. |
| 2 | Please ensure that you refer to Figure 1 and 4 in your text as, if accepted, production will need this reference to link the reader to the figure. | It has been corrected now. The number of figures has been revised now. |
| 3 | Please upload a copy of Figure 4, to which you refer in your text on page 15. If the figure is no longer to be included as part of the submission please remove all reference to it within the text. | The Fig. 4 is available and will be uploaded. It will be Fig 5 now as Fig. 5 is changed to Fig. 1. |
| 4 | We note that [Figure(s) 5] in your submission contain map/satellite images which may be copyrighted. All PLOS content is published under the Creative Commons Attribution License (CC BY 4.0), which means that the manuscript, images, and Supporting Information files will be freely available online, and any third party is permitted to access, download, copy, distribute, and use these materials in any way, even commercially, with proper attribution. For these reasons, we cannot publish previously copyrighted maps or satellite images created using proprietary data, such as Google software (Google Maps, Street View, and Earth). For more information, see our copyright guidelines:  <http://journals.plos.org/plosone/s/licenses-and-copyright>. | Fig. 5 is now changed to Fig. 1. This is basically Map of the study area. There is no issue of copy right as the map is self-generated. Therefore, there is no such issue. |
| 5 | We require you to either (1) present written permission from the copyright holder to publish these figures specifically under the CC BY 4.0 license, or (2) remove the figures from your submission: | There is no copy right issue with any figure. All the Fig. are my own generated. Therefore, there is no need to get permission from any one. |
| 6 | We note you have included a table to which you do not refer in the text of your manuscript. Please ensure that you refer to Table 3 in your text; if accepted, production will need this reference to link the reader to the Table. | It has been corrected accordingly. |
| 7 | Please include a copy of Table-4 and Table 5 which you refer to in your text on page 24 and 22. | Corrected accordingly. |
|  | We noticed you have some minor occurrence of overlapping text with the following previous publication(s), which needs to be addressed:  - <https://scialert.net/fulltext/?doi=ajps.2007.148.152>  The text that needs to be addressed involves the Introduction, the caption for Figure 1, and the final paragraph of the Discussion.  In your revision ensure you cite all your sources (including your own works), and quote or rephrase any duplicated text outside the methods section. Further consideration is dependent on these concerns being addressed. | It has been addressed. The manuscript has been proofread and where improvement was required, it has been done accordingly. |
| **Reviewer Comments to the Author “under general requirements” (Copied from email)** | | |
| **Reviewer #1:** | | |
| **S.No.** | **Reviewer’s comments** | **Author’s response** |
| 1 | The abstract is very bulky and subjective. The text in the abstract should be reduced/trimmed. | It has been revised and tried to make it short. |
| 2. | The last paragraph of the introduction section given after the objectives should be shifted to discussion. It is not appropriate to give text after stating your specific objectives. The said paragraph also needs to be supported with appropriate references. | This paragraph has been shifted to M&M section with improvements. |
| 3. | A brief detail of climate should be given in the methodology/study area section.. | It has been added in the material & method under Study area. |
| 4 | The 1st paragraph of the results section should be shifted to the methodology as it is not the results. | It has been shifted in Material & Methods section with some changes. |
| 5. | The tables and figures should be cited in the results text. | It has been improved accordingly. |
| 6. | Authors should correlate their own results to the text given in the discussion section. | It has been revised and improved. |
| 7. | The conclusion section is also bulky, just like abstract and needs reduction in bulk. | It has been trimmed accordingly. |
| **Reviewer #3** | | |
| **S.No.** | **Reviewer’s comments** | **Author’s response** |
| 1 | Please shorten the abstract. Such as it went into too many details of the informers. | It has been revised and shorten to some extent. |
| 2. | Pay attention to reference 64 in the introduction part. Aren’t they labeled by number orders? | It has been organized in ascending order. |
| 3. | Study area in the materials and methods should be clear. I do not understand the necessarity of the first sentence. The investigation was done in a small part of the Neelum Valley. Do they really represent the whole area? If not, you should point out the actual area you covered. Only about a hundred informers were collected, compared to over 1 million population. That is really a small sampling. It would be nice if the sampling area is labeled in the figure 5. | It has been improved. The study area covers most part of the district Neelum especially the high altitude areas. The population of the whole District Neelum is 1,9 million. Almost half of the population are in the study area. So the sample size is not that small if we consider the population of the area. |
| 4. | To make the best use of the information generated in this paper, I would suggest to compare the medicine with other papers that generated from nearby area in the country. | It is additional work that cannot be covered during the review process. It will be documented in the next paper. |
| **Reviewer #4** | | |
| **S.No.** | **Reviewer’s comments** | **Author’s response** |
|  | The manuscript is well written but still needs substantial revision and English correction. Author knowledge regarding the study seems to be very narrow and lacks innovative interpretation. More literature study not restricted to study area only but also the Himalaya region is the most. See attachment. | The manuscript has been revised and proof reading has been done carefully for language improvement as well. More than 65 papers and books have been consulted and referred at the end. Being native of the area, the author is quite familiar with the culture, customs and needs of the people. As I am associated with the plant sciences from last 20 years, have conducted three research projects in the same area, I am unable to respond this specific question. |
|  |  |  |
