# Supplementary material for: Ethno-veterinary practice for the treatment of animal diseases in Neelum Valley, Kashmir Himalaya, Pakistan
Source: PLoS One. 2021 Apr 30;16(4):e0250114. doi: 10.1371/journal.pone.0250114 (PMC8087047; doi:10.1371/journal.pone.0250114)
Supplement: Attachment — Submitted filename: Response to Reviewers .docx [file pone.0250114.s009.docx]

**'Response to Reviewers’**

**PONE-D-20-31879**

**ETHNO-VETERINARY PRACTICES USED FOR THE TREATMENT OF ANIMAL DISEASES IN NEELUM VALLEY, KASHMIR HIMALAYA.**

**PLOS ONE**

| **Editors Comments** | | |
| --- | --- | --- |
| **S.No.** | **Editor’s comments** | **Author’s response** |
| 1 | Which one? Recommend few! | Corrected accordingly. P. 2, L. 40 |
| 2 | You have need to rewrite Introduction on pure scientific basis. It is mandatory to keep the sequence of the text throughout the whole section.  Provide background information and set the context. Introduce your research and explain why it is worth study. Mention past attempts to solve the research problem or to answer the research question. Conclude the Introduction by mentioning the specific objectives of your research. | It has been revised thoroughly. |
| 3 | Add citations to the text. | Needful done accordingly. Text revised and Citation added P.3, L. 53,55 |
| 4 | Rewrite in a proper scientific language | Corrected accordingly. P3, L 67-71 |
| 5 | Citation please | Needful done accordingly P.4, L. 84 |
| 6 | Add few at the end of the sentence. | It has already been mentioned. No need to correct. P.5, L. 86-87 |
| 7 | Rewrite your aims and objectives which are not clear in this section. | It has been revised accordingly.  Page 6 L. 126-129 |
| 8 | Check Figures and provide figure captions/legends in the text where appropriate | Corrected accordingly  P. 7, L. 136 |
| 9 | Is this true that these herbs are dominating your study area? | Yes. These are unpalatable herbs and have develop large patches on the area where the vegetation was under severe grazing pressure. These are indicator of the disturbed vegetation as well. P.7, L. 145-146 |
| 10 | ??? | A local caste (ترک) P. 7, L. 148 |
| 11 | Write down how ethical approval was obtained? | The research protocol was finalized and the questionnaire for data collection were developed and shared with the Research Supervisory Committee (RSC) for approval. After approval from the RSC, field work was started. For ethical approval, the respondents were verbally informed about their interviews and discussions that all will be recorded and formal verbal approval was obtained on each site before data collection.P.7, L. 145-147 |
| 12 | How? No consultation of Red list? | This was done on the basis of field observations and informations collected from the inhabitants of the area. The past record about distribution of these species was obtained from the people during interviews. P.10, L. 198 |
| 13 | Stands for what? | Details added. P.15, L. 265 |
| 14 | Was the whole division your research area? | Yes. It was a complete division. |
| 15 | Don't reiterate your results. Summarize your results and their interpretation in the light of available literature. Explain the importance of your results. Acknowledge shortcomings and future directions. | It has been revised and corrected.  P.32, L.325-433 |
| 16 | Provide citation to the text and use sound English | Citations added  P.32, L. 337,343 |
| 17 | Logically describe why these families were dominating in the area | It has been improved and more clarified.  P.33, L.313-314 |
| 18 | This sentence is confusing. | Corrected accordingly  P.33, L.316 |
| 19 | Which plants? | Corrected accordingly  P. 34, L. 330 |
| 20 | Don’t abbreviate | Corrected accordingly P.35, L. 355 |
